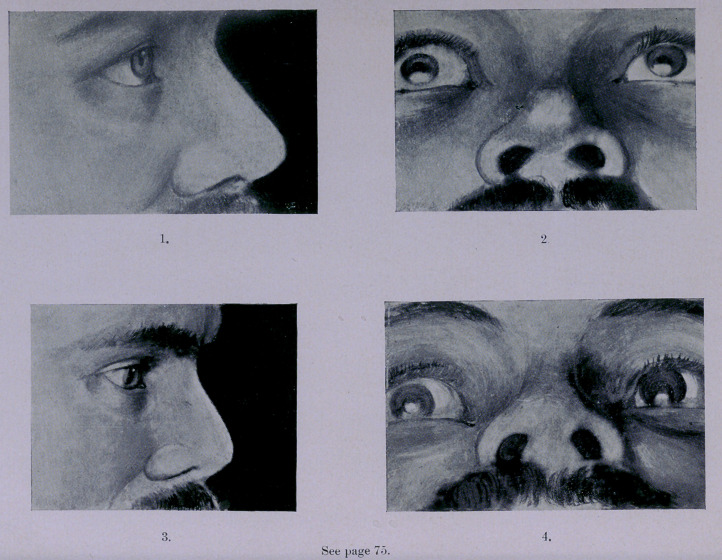# The Plate That Appears as a Frontispiece

**Published:** 1894-04

**Authors:** 


### HOMŒOPATHIC PHYSICIAN,

A MONTHLY JOURNAL OF
HOMŒOPATHIC MATERIA MEDICA AND CLINICAL MEDICINE.

"If our school ever gives up the strict inductive method of Hahnemann, we are lost, and deserve only to be mentioned as a caricature in the history of medicine."—CONSTANTINE HERING.

Vol. XIV. APRIL, 1894. No. 4.

### EDITORIALS.

THE PLATE THAT APPEARS AS A FRONTISPIECE in this number was originally intended to illustrate the surgical case performed by Dr. Edmund Carleton, of New York, and described by him in the March number of this journal at page 75.

By an oversight of the printer, the number was made up and mailed to our subscribers without it. It is, therefore, reproduced here so that when the volume for the year is bound, it may ultimately find its proper place.

## Figures and Tables

**Figure f1:**